# Fed-DTCN: A Federated Disentangled Learning Framework for Unsupervised Zero-Day Anomaly Detection in IoT with Semantic-Aware Augmentation

**DOI:** 10.3390/s26061918

**Published:** 2026-03-18

**Authors:** Muhammad Ali Khan, Osman Khalid, Rao Naveed Bin Rais

**Affiliations:** 1 Department of Computer Science, COMSATS University Islamabad, Abbottabad Campus, Abbottabad 22060, Pakistan; malikhan@cuiatd.edu.pk (M.A.K.); osman@cuiatd.edu.pk (O.K.); 2 Artificial Intelligence Research Center, Ajman University, Ajman 346, United Arab Emirates

**Keywords:** anomaly detection, contrastive learning, federated learning, Internet of Things, non-IID, zero-day attacks

## Abstract

The proliferation of Internet of Things (IoT) devices continues to expand the network attack surface while introducing stringent privacy requirements that challenge effective intrusion detection. Federated learning enables collaborative model training without centralizing raw network telemetry. However, existing federated intrusion detection approaches often degrade under statistical heterogeneity and remain vulnerable to zero-day attacks when they rely on labeled data or reconstruction-based objectives. This work proposes Fed-DTCN (Federated Dual Temporal Contrastive Network), an unsupervised federated framework for zero-day anomaly detection in IoT environments. Fed-DTCN learns robust representations of benign IoT traffic using contrastive learning with semantic-preserving augmentations. A dual-encoder architecture disentangles globally shared features from client-specific patterns, improving generalization under heterogeneous federated deployments. Personalization and privacy are preserved by selectively aggregating only the shared encoder parameters. The framework employs a compact temporal convolutional backbone together with a soft-weighted contrastive objective to constrain benign representations, thereby enabling reliable detection of out-of-distribution threats. Extensive experiments on the TON_IoT and CSE-CIC-IDS2018 benchmarks show that Fed-DTCN matches or surpasses a state-of-the-art supervised baseline on standard attacks, achieving an F1-score of 99.99% on TON_IoT. In a zero-day evaluation where the Botnet class is withheld during training, Fed-DTCN attains an F1-score of 96%, compared to 0.52% for the supervised baseline. Ablation studies validate the effectiveness of the proposed augmentations, while evaluations under heterogeneous client partitions demonstrate reduced inter-client variance and consistent per-client improvements, indicating suitability for realistic IoT deployments.

## 1. Introduction

Internet of Things (IoT) infrastructures have become a foundational layer of modern cyber-physical systems, connecting vast numbers of resource-constrained devices across heterogeneous and often untrusted environments. Billions of heterogeneous devices now operate at the network edge, continuously generating high-dimensional telemetry across diverse environments. Industry projections estimate that more than 39 billion IoT endpoints will be active by 2030 [1], spanning critical sectors such as industrial control systems, smart cities, and healthcare infrastructures. While this proliferation enables fine-grained monitoring and automation, it also substantially enlarges the attack surface. Large-scale incidents, including Mirai-family botnets and stealthy infiltration campaigns, have demonstrated that compromised IoT devices can be leveraged to launch persistent and disruptive attacks [2]. Consequently, anomaly-based intrusion detection at distributed gateways has emerged as a critical security requirement. For clarity, we distinguish between the terms intrusion detection and anomaly detection. Intrusion detection refers to the broader network security task of identifying malicious activity in network traffic, whereas anomaly detection denotes a learning paradigm that identifies deviations from normal behavior. In this work, anomaly detection serves as the underlying mechanism for intrusion detection, enabling the detection of previously unseen threats without requiring labeled attack data.

Despite its importance, practical intrusion detection in IoT environments faces several systemic challenges. First, raw traffic data is inherently sensitive and subject to regulatory and organizational constraints that preclude centralized collection. Second, traffic characteristics vary widely across deployment sites due to differences in device populations, protocol usage, and operational patterns, leading to pronounced non-independent and identically distributed (non-IID) behavior. Third, labeled attack data is typically scarce, incomplete, or entirely unavailable in operational settings, particularly for emerging zero-day threats. Together, these factors severely limit the applicability of conventional supervised learning approaches and post-hoc thresholding strategies, which implicitly assume access to representative labeled attacks.

Federated learning (FL) offers a natural architectural response to these constraints by enabling collaborative model training without centralizing raw telemetry [3]. However, existing FL-based intrusion detection systems remain inadequate for realistic IoT deployments. Prior empirical analyses of centralized and federated IDS under data and label skew report substantial performance degradation in heterogeneous settings [4,5]. Supervised federated approaches rely on labeled attack samples and consequently exhibit poor generalization to previously unseen threat families, due to their dependence on closed-set decision boundaries learned from historical attack distributions [6]. Reconstruction-based methods, while label-free, frequently entangle benign client-specific behavior with anomalous deviations, leading to elevated false-positive rates under heterogeneous conditions, because non-IID variations across clients distort the learned notion of global normality [7]. Other approaches that depend on public proxy datasets, cross-client embedding exchange, or centralized alignment mechanisms introduce additional privacy risks and increase deployment complexity, due to reliance on shared data or representation exposure beyond local clients [8,9], thereby undermining the core motivations of federated learning.

Beyond the federated setting, representation learning for network traffic presents its own challenges. Many centralized contrastive and temporal models employ generic data augmentations such as random cropping, shuffling, or aggressive temporal distortion [10,11,12]. When applied to network traffic, these operations can violate protocol causality and distort the semantics of communication flows. Some methods further rely on computationally intensive preprocessing, including dynamic time warping or causal discovery [3,13,14], rendering them impractical for resource-constrained edge gateways. Moreover, standard instance-level contrastive objectives implicitly treat all non-positive samples as equally dissimilar [10], an assumption that breaks down in benign-dominated regimes where semantically consistent traffic patterns may appear superficially different [11,12]. Collectively, these limitations expose a clear gap in existing solutions.

Based on limitations in prior work and the constraints of realistic federated IoT deployments, an effective intrusion detection framework should satisfy four key requirements: (i) operate in an unsupervised, benign-only training setting to enable zero-day detection when labels are limited; (ii) preserve protocol-level causal semantics during representation learning to avoid distorting communication behavior; (iii) disentangle globally shared invariants from client-specific patterns to mitigate non-IID effects in federated training; and (iv) remain lightweight and privacy-preserving to support deployment on resource-constrained edge devices. Existing approaches address some of these requirements in isolation, but do not satisfy them jointly.

To address this gap, we introduce Fed-DTCN (Federated Dual Temporal Contrastive Network), an unsupervised federated learning framework for zero-day anomaly detection in IoT networks. Fed-DTCN adopts a dual-encoder architecture built on compact Temporal Convolutional Networks (TCNs), comprising a shared encoder that captures globally invariant benign patterns and a private encoder that models client-specific behaviors. Training is performed via contrastive learning with semantic-aware, causality-preserving augmentations tailored to network traffic, including causal time warping, volumetric scaling, stochastic feature perturbation, and protocol-aware masking. To mitigate erroneous repulsion between semantically similar benign samples, Fed-DTCN employs a lightweight, soft-weighted contrastive objective that leverages a frozen auxiliary projector for affinity estimation. An explicit orthogonality regularizer further enforces decorrelation between shared and private representations, enabling selective aggregation of shared parameters while preserving personalization and privacy.

During inference, the learned representations support both global and client-adaptive anomaly scoring through centroid-based similarity measures, allowing deployments to balance sensitivity to universal attack patterns with robustness to local operational variations. Extensive experiments on diverse IoT and network security benchmarks demonstrate that Fed-DTCN matches or exceeds supervised baselines on standard attacks and achieves strong zero-day detection performance when evaluated on previously unseen attack classes. In particular, when the Botnet class in CSE-CIC-IDS2018 is withheld during training, Fed-DTCN achieves an F1-Score of 96%, whereas a state-of-the-art supervised federated baseline attains 0.52%. Additional ablation studies confirm the semantic value of the proposed augmentations, while evaluations under non-IID partitions show reduced inter-client variance and consistent per-client improvements, indicating suitability for heterogeneous federated deployments.

The main contributions of this work are summarized as follows:

- We propose Fed-DTCN, a fully unsupervised federated framework for zero-day anomaly detection that learns representations of normal network traffic without relying on labeled attacks or reconstruction-based objectives.
- We design semantic-aware, causality-preserving data augmentations tailored to network traffic, enabling effective contrastive learning while maintaining protocol semantics.
- We introduce a dual-encoder architecture with an orthogonality regularizer to disentangle shared invariants from client-specific variations, addressing statistical heterogeneity and enhancing privacy through selective aggregation.
- We develop a soft-weighted contrastive objective that reduces erroneous repulsion among semantically consistent benign samples in benign-dominated regimes.
- We show through extensive experiments that Fed-DTCN provides strong zero-day generalization (96% F1-Score with a withheld Botnet class) and consistently lower inter-client variance across IID and non-IID federated partitions.

The remainder of this paper is organized as follows. Section 2 reviews related work. Section 3 formalizes the problem and the threat model. Section 4 presents the proposed Fed-DTCN methodology. Section 5 reports experimental results and ablation studies. Section 6 concludes with limitations and deployment considerations.

## 2. Related Work

This section reviews prior research most relevant to Fed-DTCN, focusing on three complementary areas: (i) federated learning for intrusion detection, (ii) contrastive representation learning for time-series and network security, and (iii) disentanglement and augmentation strategies for handling statistical heterogeneity. For each area, we discuss representative methods and critically examine their limitations in the context of unsupervised, zero-day intrusion detection under privacy and edge-deployment constraints.

### 2.1. Federated Learning for IoT Intrusion Detection

FL enables collaborative model training across distributed clients without centralizing raw telemetry and has therefore been widely investigated for privacy-preserving intrusion detection in IoT environments. Early FL-based IDSs typically combine the FedAvg [15] aggregation strategy with local classifiers or reconstruction-based models such as autoencoders. As shown by Sun et al. [16] and Nguyen et al. [17], these approaches often suffer from elevated false-positive rates and unstable global convergence when local traffic distributions are highly imbalanced or non-IID.

More recent studies incorporate representation learning into federated IDS frameworks, but several limitations remain for zero-day detection. The authors of FeCo [6] integrate contrastive objectives into FL to improve separability between benign and malicious flows. However, FeCo [6] relies on supervised contrastive learning and therefore requires labeled attack samples, which are rarely available in realistic deployments and fundamentally limit generalization to unseen threat families. Dong et al. [8] propose FADngs, which aligns client distributions by exchanging noisy density statistics and performing self-supervised distillation using a public proxy dataset. While effective under controlled settings, this approach depends on representative public data and cross-domain augmentations, reducing its applicability to proprietary or industrial IoT traffic. Kong et al. [9] introduce FedCAD, a graph-based federated framework that addresses rare-node scenarios through embedding exchange; although beneficial in specific contexts, transmitting embeddings increases communication overhead and raises privacy concerns for high-throughput network streams.

Other federated IDS designs highlight additional trade-offs. FedAware [7] employs reconstruction-driven aggregation heuristics, but reconstruction error can be inflated by benign, client-specific behavior, resulting in increased false alarms under heterogeneity. Privacy-enhanced FL frameworks based on cryptographic primitives, such as homomorphic encryption [18], provide stronger formal guarantees but incur substantial computational overhead, making them impractical for latency-sensitive edge gateways. Recent studies have also examined the limitations of FL for intrusion detection under heterogeneous IoT environments. In prior work, Khan et al. [4,5] conducted a comparative evaluation of centralized and federated intrusion detection models under data- and label-skew conditions. Their analysis showed that supervised federated IDS models can suffer from degraded detection performance and unstable convergence when client traffic distributions are highly non-IID and attack labels are unevenly distributed. These findings highlight the challenges of applying conventional supervised FL frameworks to intrusion detection in realistic IoT deployments. The present work builds on these observations by exploring an unsupervised federated representation learning approach designed to better accommodate heterogeneous client traffic. In summary, most existing federated IDS approaches either require labeled attacks, depend on external or public datasets, or exchange intermediate representations. Fed-DTCN avoids these constraints by operating in a benign-only unsupervised regime and by aggregating only shared encoder parameters.

### 2.2. Contrastive Representation Learning for Time-Series and Network Security

Recent deep learning approaches have explored hybrid architectures combining convolutional feature extraction, recurrent modeling, and attention mechanisms for intrusion detection. The authors in [19] propose an attention-enhanced BiLSTM–ANN framework with CNN-based feature selection to improve classification performance on labeled intrusion datasets. By integrating feature-selection layers with sequence modeling, the method achieves strong detection accuracy in centralized training environments. However, the approach relies on supervised learning and centralized data collection, which limits its applicability to privacy-preserving IoT deployments and benign-only zero-day detection scenarios. In addition, recurrent–attention architectures introduce higher computational overhead compared with lightweight temporal convolutional models, making deployment on resource-constrained edge gateways challenging. Contrastive learning has emerged as a powerful paradigm for time-series representation learning and anomaly detection. Yue et al. [10] propose TS2Vec, and related dilated-convolutional models learn multi-scale temporal representations but typically rely on generic augmentations and explicit negative mining. In network traffic analysis, such operations can violate packet-level causality and distort protocol semantics. To address false repulsion among similar samples, Lee et al. [11] propose SoftCLT, which introduces hardness-aware weighting into contrastive objectives; however, several implementations rely on computationally expensive sequence-alignment procedures such as dynamic time warping (DTW), rendering them unsuitable for high-frequency edge deployments.

A number of studies design contrastive objectives specifically for anomaly detection. CARLA [12] injects synthetic anomalies as explicit negatives to shape decision boundaries, improving detection of modeled anomaly types but potentially biasing the learned representation toward injected templates and reducing generalization to unforeseen attacks. TS-TCC [20] applies permutation-based augmentations and cross-view prediction to learn temporal features; however, its aggressive reordering operations disrupt protocol causality and are therefore ill-suited for packet-ordered network traffic. FreRA [21] explores frequency-domain masking to generate contrastive views, but spectral perturbations may fail to preserve the temporal dependencies required for protocol-level intrusion detection. ContraMTD [22] enforces host-interaction consistency using graph constraints, which benefits certain host-level tasks but relies on centralized topology information and graph construction.

Transformer- and attention-based architectures, including MPFormer [23] and DCdetector [24], achieve strong centralized detection performance but incur quadratic complexity in sequence length and high memory consumption, limiting their practicality on resource-constrained gateways. Similarly, approaches that transform time series into image-like modalities or employ extensive causal discovery introduce modality artifacts or heavy preprocessing pipelines [13,25], which conflict with edge deployment requirements. Overall, the contrastive learning literature provides valuable algorithmic components, such as instance discrimination and hardness-aware weighting, but also exposes two persistent challenges for federated zero-day detection: commonly used augmentations may violate network causality, and many robustness techniques require expensive computation or cross-client data exchange. Fed-DTCN addresses these challenges by employing protocol-aware, order-preserving augmentations, a lightweight frozen auxiliary projector for semantic weighting, and fully local contrastive optimization with selective aggregation.

### 2.3. Disentanglement, Heterogeneity, and Augmentation Design

Explicitly disentangling shared and client-specific factors is a common strategy for mitigating heterogeneity in federated learning. For example, the authors of FDFL [26] decompose representations into global and local components to reduce negative transfer and enable personalization. Similarly, MOON (Model-Contrastive Federated Learning) [27] addresses statistical heterogeneity by introducing a model-level contrastive objective that encourages local representations to remain consistent with the global model during federated training, improving stability under non-IID client distributions. Probabilistic and variational disentanglement methods, such as SC-STVAE [28] and DCVAE [14], provide expressive modeling capabilities but often rely on reconstruction objectives or supervised causal annotations. In the context of anomaly detection, reconstruction-driven losses can inadvertently incorporate rare or anomalous patterns into the nominal manifold, thereby degrading detection sensitivity.

Graph-based and multimodal disentanglement approaches, including GEM [29] and MGCLAD [30], offer alternative perspectives but typically require centralized graph construction, access to global topology, or cloud-scale models. These assumptions conflict with the latency, computational, and privacy constraints of edge IoT deployments. Augmentation design presents a related challenge: naive permutations or vision-inspired transformations commonly used in time-series contrastive learning disrupt packet ordering and protocol semantics, while synthetic anomaly injection biases the learned decision boundary toward modeled attacks.

Fed-DTCN integrates semantically valid, causality-preserving augmentations with an explicit dual-encoder disentanglement objective. Rather than relying on reconstruction losses, the proposed framework employs contrastive alignment in projection spaces together with an orthogonality regularizer between shared and private subspaces. This design mitigates negative transfer while avoiding labeled attacks, public proxy datasets, and computationally expensive causal discovery procedures.

Summary of Gaps. Table 1 summarizes representative intrusion detection and time-series anomaly detection approaches across several design dimensions relevant to federated zero-day detection, including learning paradigm, architecture, representation learning strategy, representation structure, and aggregation mechanism. The comparison highlights a critical gap at the intersection of unsupervised federated learning, protocol-preserving augmentation, and lightweight disentanglement suitable for edge deployments. While prior methods address subsets of these challenges, Fed-DTCN integrates benign-only training, protocol-aware augmentations, explicit representation disentanglement, and communication-efficient federated aggregation within a unified framework. Fed-DTCN is designed to fill this gap by (i) learning a benign traffic manifold using protocol-aware augmentations, (ii) disentangling global and client-specific factors through selective aggregation and orthogonality regularization, and (iii) preserving privacy by transmitting only shared encoder updates.

## 3. Threat Model and Problem Formulation

### 3.1. Threat Model

We consider a network-level adversary targeting an IoT infrastructure by injecting previously unseen and stealthy attack traffic that complies with standard communication protocols. The adversary’s objective is test-time evasion: to generate malicious network flows $x_{\mathrm{adv}}$ whose statistical and protocol-level characteristics partially overlap with benign traffic, thereby reducing detectability while preserving functional attack semantics.

The attacker operates under an open-set (zero-day) setting. Entire attack families drawn from an unknown distribution $Q_{\mathrm{attack}}$ are absent from the training data and appear only during inference. Formally, test samples are drawn from $Q_{\mathrm{attack}}$ such that $\mathrm{supp}\left(Q_{\mathrm{attack}}\right)\cap \mathrm{supp}\left(P_{\mathrm{benign}}\right)\neq \emptyset$, while $Q_{\mathrm{attack}}\cap P_{\mathrm{train}}=\emptyset$.

The adversary is assumed to be black-box with respect to the learning system and has no access to model parameters, internal representations, training data, or semantic augmentation policies. Its interaction with the system is limited to generating network traffic at test time.

The scope of this work is restricted to evasion under semantic distribution shift. Attacks against the federated learning process itself, including Byzantine behavior, data poisoning, model poisoning, and backdoor insertion, are outside the scope of this study. The federated infrastructure is therefore assumed to execute the training protocol correctly, with all clients following the prescribed optimization procedure.

Privacy Assumptions. In addition to the external network adversary described above, we assume the system follows an honest-but-curious server model in which the server correctly executes the aggregation protocol but may attempt to infer information from received model updates. Prior work has shown that parameter sharing in FL may expose systems to risks, such as gradient leakage or model inversion attacks. In Fed-DTCN, only shared encoder updates are transmitted during aggregation, while client-specific private encoder parameters remain strictly local, reducing the amount of information exposed to the server. However, the proposed framework does not implement formal cryptographic protections such as secure aggregation or differential privacy, and defending against such inference attacks is outside the scope of this work.

This threat model is consistent with the experimental protocol adopted in the evaluation, in which entire attack categories are excluded during training and introduced only at test time.

### 3.2. Problem Formulation

We consider a federated IoT environment comprising *K* edge gateways (clients), denoted by $C=\{C_{1},\ldots ,C_{K}\}$. Each client $C_{k}$ observes a private and continuous stream of multivariate network traffic, represented as a local dataset $D_{k}={\left\{x_{i}^{\left(k\right)}\right\}}_{i=1}^{n_{k}}$. Each sample $x\in R^{T\times F}$ corresponds to a traffic window of length *T* time steps with *F* features.

For clarity and to support semantic-aware augmentation, each traffic window is decomposed along the feature axis as

$$x=[x_{\mathrm{id}},\;x_{f}],$$

where $x_{\mathrm{id}}$ captures identity- or header-related attributes (e.g., protocol fields and device identifiers), while $x_{f}$ represents behavioral flow statistics such as packet counts, byte volumes, and inter-arrival times. Throughout the remainder of the paper, x denotes a complete traffic window, while $x_{\mathrm{id}}$ and $x_{f}$ are referenced explicitly when augmentation operations are applied selectively. The global dataset is defined as $D={⋃}_{k=1}^{K}D_{k}$, with a total number of training samples $N_{\mathrm{tot}}=\sum_{k=1}^{K}n_{k}$. The main notations used in this paper are in Table 2.

The anomaly detection task is formulated under three fundamental constraints. First, the system operates in an unsupervised zero-day setting: the training dataset $D_{\mathrm{train}}\subset D$ contains only benign traffic, while anomalies originate from unseen attack distributions $Q_{\mathrm{attack}}$ that are disjoint from the training support. Second, the federated environment exhibits pronounced statistical heterogeneity, as client data are drawn from distinct benign distributions $P^{\left(k\right)}$, with $P^{\left(i\right)}\neq P^{\left(j\right)}$ in general. This non-IID property must be explicitly addressed to prevent performance degradation of the global model. Third, the representation learning process must preserve semantic validity: any augmentation t(x) used to generate contrastive views must maintain the causal semantics of the original traffic to ensure physically meaningful invariances.

To satisfy these requirements, the federated system maintains a global shared parameter set $\Theta_{s}$, while each client maintains a local parameter set $\left\{\theta_{s}^{\left(k\right)},\theta_{p}^{\left(k\right)}\right\}$. Here, $\theta_{s}^{\left(k\right)}$ denotes the local instance of the globally shared parameters synchronized from the server, and $\theta_{p}^{\left(k\right)}$ represents client-specific private parameters. The federated optimization objective is formulated as

(1) $$\min_{\Theta_{s},\left\{\theta_{p}^{\left(k\right)}\right\}}\frac{1}{N_{\mathrm{tot}}}\sum_{k=1}^{K}n_{k}E_{x∼P^{\left(k\right)}}\left[L\left(x;\Theta_{s},\theta_{p}^{\left(k\right)}\right)\right].$$

This objective follows the standard federated empirical risk minimization formulation [15], where the shared parameters $\Theta_{s}$ are optimized across clients while the private parameters $\theta_{p}^{\left(k\right)}$ remain client-specific.This optimization is subject to strict privacy and communication constraints: raw data $D_{k}$ never leave the local client, and only the local copies of the shared parameters $\theta_{s}^{\left(k\right)}$ are synchronized with the central server. Private parameters $\theta_{p}^{\left(k\right)}$ remain local to absorb client-specific variability. After convergence, each client computes an anomaly score $A^{\left(k\right)}\left(x\right)$ designed to maximize detection probability for zero-day attacks,

$$\underset{x∼Q_{\mathrm{attack}}}{\mathrm{Pr}}\left[A^{\left(k\right)}\left(x\right)>\rho^{\left(k\right)}\right],$$

while ensuring a bounded false-positive rate on benign traffic,

$$\underset{x∼P^{\left(k\right)}}{\mathrm{Pr}}\left[A^{\left(k\right)}\left(x\right)>\rho^{\left(k\right)}\right]\leq \alpha .$$

## 4. Methodology

**Figure 1:**
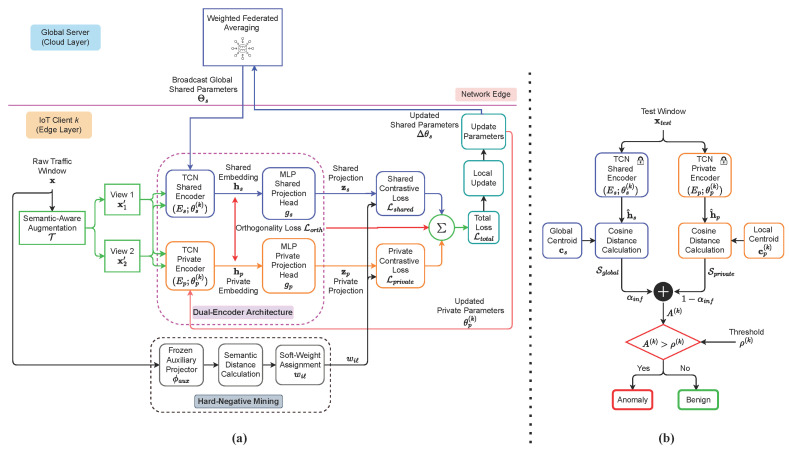
Overview of the proposed Fed-DTCN framework. (**a**) Federated training phase: Each client trains a dual-encoder model consisting of a shared encoder $E_{s}$ and a private encoder $E_{p}$ using local benign traffic. The learning objective enforces contrastive alignment while promoting representation disentanglement through an orthogonality regularizer. Only updates to the shared parameters $\Delta \theta_{s}^{\left(k\right)}$ are communicated to the server for weighted aggregation. (**b**) Local inference phase: Incoming traffic windows are encoded using the trained encoders to obtain normalized representations, which are compared against pre-computed benign centroids. An anomaly score $A^{\left(k\right)}$ is computed via similarity-based fusion and evaluated against a client-specific threshold $\rho^{\left(k\right)}$ to detect anomalous behavior.

This section presents Fed-DTCN, a federated and unsupervised framework for zero-day anomaly detection in IoT systems. The proposed methodology is structured around four tightly integrated components: (i) semantic-aware causal augmentations, (ii) a dual-encoder architecture that disentangles globally shared and client-specific representations, (iii) a soft-weighted contrastive learning objective, and (iv) an orthogonality regularizer that explicitly enforces representational decorrelation. All components are designed to operate under strict privacy constraints and pronounced statistical heterogeneity across clients. An overview of the complete training and inference pipeline is illustrated in Figure 1.

The figure distinguishes between the federated training phase, where shared representations are learned collaboratively across clients, and the local inference phase, where anomaly detection is performed independently at each client using frozen encoders.

### 4.1. Semantic-Aware Causal Augmentations

Let T denote a family of augmentation operators designed to preserve protocol-level causal semantics. Following the formulation introduced in the problem definition, each input traffic window x is decomposed into identity features $x_{\mathrm{id}}$ and behavioral flow metadata $x_{f}$. This decomposition enables augmentation operators to act selectively on semantically distinct subspaces without violating protocol constraints or causal ordering.

Throughout this section, the full traffic window is denoted by x, while $x_{\mathrm{id}}$ and $x_{f}$ are referenced explicitly only when an augmentation targets a specific feature subspace. For each input x, two correlated augmented views $x_{1}^{\prime }$ and $x_{2}^{\prime }$ are generated locally at the client by sampling distinct but complementary pipelines from T(x).

**Figure 2:**
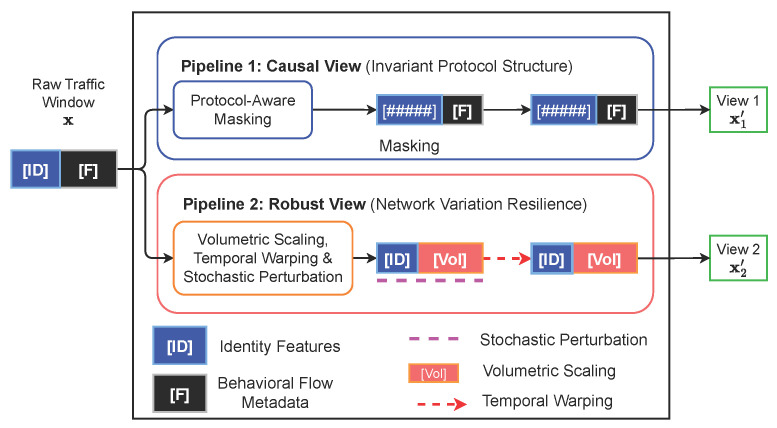
Semantic-aware augmentation module T. Each input traffic window x is partitioned into identity/header features $x_{\mathrm{id}}$ and behavioral flow features $x_{f}$. The first augmentation pathway produces a causality-preserving view by masking identity-related attributes to emphasize globally shared invariants. The second pathway generates a robustness-oriented view by applying controlled perturbations to flow-level features, modeling benign temporal and volumetric variability commonly observed in real network traffic.

Unlike generic augmentation strategies commonly adopted in computer vision, all operators in T are explicitly constrained to preserve the physical semantics and causal structure of network traffic. The augmentation pipelines are summarized in Figure 2 and described as follows:

- Protocol-aware masking (Pipeline 1): Structured masking is applied to contiguous protocol fields within the identity subspace $x_{\mathrm{id}}$. By selectively obscuring fixed header attributes such as IP addresses and device identifiers, this operation suppresses shortcut learning based on site-specific information and encourages the shared encoder to extract invariant protocol-level patterns.
- Volumetric scaling (Pipeline 2): Volume-related features in the behavioral metadata subspace $x_{f}$, including packet counts and byte totals, are scaled by a multiplicative factor γ∼Uniform[1−ϵ,1+ϵ]. This augmentation simulates benign traffic burstiness while preserving the intrinsic behavioral manifold relevant for detecting volumetric anomalies.
- Causal time warping: Temporal variability is introduced through a smooth, monotonic time-warping function $t^{\prime }=\psi \left(t\right)$ implemented via spline interpolation, where $\psi^{\prime }\left(t\right)>0$. This operation perturbs packet inter-arrival times while strictly preserving event ordering, thereby maintaining protocol causality.
- Stochastic feature perturbation: Fine-grained additive noise δ is injected into continuous features of $x_{f}$. For each feature $x_{t,f}$, the perturbed value is ${\tilde{x}}_{t,f}=x_{t,f}+\delta_{t,f}$, where $\delta_{t,f}∼N\left(0,\sigma_{f}^{2}\right)$. The noise magnitude $\sigma_{f}$ is calibrated using robust interquartile ranges to reflect realistic sensor uncertainty.

Collectively, these augmentation pipelines expand the benign data manifold while preserving semantic validity and causal coherence. This design ensures that the learned representations remain invariant to operational fluctuations commonly observed in real-world IoT traffic, which is essential for robust zero-day anomaly detection. The effectiveness of these semantic-aware augmentation operators is empirically validated in the ablation analysis presented in Section 5.8.1, where replacing the proposed transformations with unconstrained perturbations significantly degrades anomaly detection performance, demonstrating the importance of preserving protocol semantics during augmentation.

### 4.2. Dual Encoder Architecture

To explicitly disentangle global invariants from client-specific variations, each client maintains two backbone encoders: a shared encoder $E_{s}\left(\cdot ;\theta_{s}^{\left(k\right)}\right)$ and a private encoder $E_{p}\left(\cdot ;\theta_{p}^{\left(k\right)}\right)$. The parameters $\theta_{s}^{\left(k\right)}$ of the shared encoder are aggregated across clients via federated learning, whereas the private parameters $\theta_{p}^{\left(k\right)}$ remain local to client *k* and capture client-specific characteristics.

For an augmented input $x^{\prime }$, the encoders produce backbone embeddings

(2) $$h_{s}=E_{s}\left(x^{\prime };\theta_{s}^{\left(k\right)}\right)\in R^{d_{s}},\;h_{p}=E_{p}\left(x^{\prime };\theta_{p}^{\left(k\right)}\right)\in R^{d_{p}}.$$

Each embedding is subsequently passed through a lightweight projection head,

(3) $$z_{s}=g_{s}\left(h_{s}\right)\in R^{m_{s}},\;z_{p}=g_{p}\left(h_{p}\right)\in R^{m_{p}}.$$

The canonical dimensionalities used in all experiments are reported later in Section 5. Projection outputs are $\ell_{2}$-normalized and used exclusively for contrastive learning. Gradients propagate through the projection heads into the backbone encoders during training, while the backbone embeddings $h_{s}$ and $h_{p}$ are reserved for orthogonality regularization and inference-time anomaly scoring.

### 4.3. Soft-Weighted Contrastive Objective

**Figure 3:**
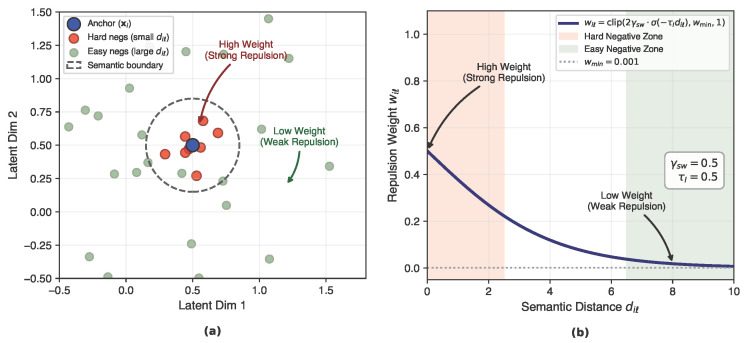
Hardness-aware soft-weighting mechanism used in the contrastive objective. (**a**) Conceptual illustration of the latent representation space induced by the auxiliary projector $\phi_{\mathrm{aux}}$, where semantically similar benign samples are identified. Pairs within closer semantic proximity are assigned higher repulsion weights to emphasize hard negatives, while distant pairs receive lower weights to limit the influence of non-informative samples. (**b**) Distance-aware weighting function that maps semantic distances $d_{i\ell }$ to soft repulsion weights $w_{i\ell }$, enabling adaptive emphasis on informative negative pairs while enforcing a lower bound $w_{\min}$ for numerical stability.

To prevent representation collapse and encourage uniformity among semantically related benign samples, we introduce a soft-weighted contrastive learning objective that modulates negative sample contributions based on semantic proximity. Semantic proximity is estimated using a frozen auxiliary projector $\phi_{\mathrm{aux}}:R^{T\times F}\to R^{d_{\mathrm{aux}}}$, implemented as a lightweight MLP operating on flattened input windows. This auxiliary projector is used solely for affinity estimation and is never updated during federated training. The conceptual mapping and distance-aware weighting mechanism are illustrated in Figure 3.

For a mini-batch of *B* anchors, we construct a flattened view set V={1,…,2B} containing both augmented views of each anchor. Each view $x_{t}^{\prime }$ is mapped to an auxiliary embedding $e_{t}=\phi_{\mathrm{aux}}\left(x_{t}^{\prime }\right)$, and pairwise distances are computed as

(4) $$d_{i\ell }={∥e_{i}-e_{\ell }∥}_{2},\;i,\ell \in \left\{1,\ldots ,2B\right\}.$$

Soft weights are assigned to negative samples according to

(5) $$w_{i\ell }=\mathrm{clip}\left(2\gamma_{sw}\cdot \sigma \left(-\tau_{I}d_{i\ell }\right),\;w_{\min},\;1\right),\;i\neq \ell ,$$

Equation (5) assigns a soft repulsion weight to each negative pair based on their distance in the auxiliary embedding space, allowing semantically similar negatives to exert stronger contrastive pressure. Here, σ(·) denotes the sigmoid function. These weights emphasize semantically close (hard) negatives while suppressing distant, non-informative samples.

The shared and private contrastive losses are defined symmetrically using the normalized projection vectors, resulting in $L_{\mathrm{shared}}$ and $L_{\mathrm{private}}$, respectively. For a positive pair of augmented views $\left(z_{i},z_{j}\right)$, the contrastive objective is defined by the soft-weighted negative log-likelihood:

(6) $$L_{i,j}=-\log\left(\frac{\exp(\mathrm{sim}\left(z_{i},z_{j}\right)/\tau )}{\exp\left(\mathrm{sim}\left(z_{i},z_{j}\right)/\tau \right)+\sum_{\ell \in V\setminus \{i,j\}}w_{i\ell }\exp\left(\mathrm{sim}\left(z_{i},z_{\ell }\right)/\tau \right)}\right)$$

This formulation extends the standard InfoNCE contrastive objective [31] by incorporating soft weights $w_{i\ell }$ for negative samples. Here, τ is the temperature parameter and sim(·) denotes the cosine similarity between $\ell_{2}$-normalized projections. The final shared and private contrastive losses, $L_{\mathrm{shared}}$ and $L_{\mathrm{private}}$, are computed by averaging these contributions over all 2B views in the mini-batch, where *j* is the index of the positive counterpart for each anchor *i*:

(7) $$\begin{array}{cc} L_{\mathrm{shared}} & =\frac{1}{2B}\sum_{i=1}^{2B}L_{i,j}\left(z_{s}\right),\\ L_{\mathrm{private}} & =\frac{1}{2B}\sum_{i=1}^{2B}L_{i,j}\left(z_{p}\right). \end{array}$$

This ensures that the learned benign manifold is constrained effectively, facilitating reliable detection of out-of-distribution zero-day threats while remaining robust to benign operational variations.

### 4.4. Orthogonality Regularization

To explicitly decouple shared and private representations, we impose an orthogonality constraint on the backbone embeddings. Let ${\bar{H}}_{s}$ and ${\bar{H}}_{p}$ denote the column-wise mean-centered and $\ell_{2}$-normalized batch matrices constructed from the flattened set of augmented views. The orthogonality loss is defined as

(8) $$L_{\mathrm{orth}}=\frac{1}{d_{s}d_{p}}{∥{\bar{H}}_{s}^{⊤}{\bar{H}}_{p}∥}_{F}^{2}.$$

This regularizer penalizes correlations between shared and private embeddings, encouraging the two representation spaces to capture complementary information.

### 4.5. Local Objective and Optimization

The total objective minimized locally at client *k* is

(9) $$L_{\mathrm{total}}^{\left(k\right)}=L_{\mathrm{shared}}+L_{\mathrm{private}}+\lambda_{\mathrm{orth}}L_{\mathrm{orth}}.$$

This objective combines the shared contrastive loss, the client-specific private contrastive loss, and an orthogonality regularization term weighted by $\lambda_{\mathrm{orth}}$ to encourage disentanglement between the two representation spaces. All loss components are optimized jointly during local training. Soft negative weights are computed once per mini-batch and reused across both contrastive branches to improve computational efficiency.

### 4.6. Federated Training with Selective Aggregation

Federated optimization proceeds over synchronous communication rounds. At each round *r*, the central server selects a subset of participating clients $S_{r}\subseteq C$ and broadcasts the current shared encoder parameters $\Theta_{s}^{r}$ to the selected clients. In the experimental configuration described in Section 5.4, all clients participate in each round (i.e., $S_{r}=C$). Each participating client performs local optimization on its private dataset, updating both the shared encoder parameters and its client-specific private encoder parameters.

After local training, each client $C_{k}\in S_{r}$ transmits only the updates to the shared encoder parameters, denoted by $\Delta \theta_{s}^{\left(k,r\right)}$, back to the server, while the private encoder parameters $\theta_{p}^{\left(k\right)}$ remain strictly local and are never communicated. This design enables personalization by allowing client-specific representations to adapt to local traffic characteristics while preserving privacy.

The server aggregates the received shared updates using a sample-size-weighted federated averaging scheme (FedAvg) rule [15]:

(10) $$\Theta_{s}^{r+1}=\Theta_{s}^{r}+\sum_{k\in S_{r}}\frac{n_{k}}{\sum_{j\in S_{r}}n_{j}}\Delta \theta_{s}^{\left(k,r\right)},$$

where $n_{k}$ denotes the number of local samples held by client $C_{k}$. By aggregating only the shared encoder parameters while preserving private components locally, the selective aggregation mechanism enables the global model to capture invariant representations across heterogeneous clients while maintaining client-level specialization.

### 4.7. Inference and Anomaly Scoring

During inference, an incoming traffic window x is encoded by the frozen shared and private backbones. Each client computes a global centroid $c_{s}$ and a client-specific centroid $c_{p}^{\left(k\right)}$ from a held-out benign calibration set. Cosine similarity scores are computed as

(11) $$S_{s}\left(x\right)=\cos\left({\hat{h}}_{s},c_{s}\right),\;S_{p}^{\left(k\right)}\left(x\right)=\cos\left({\hat{h}}_{p},c_{p}^{\left(k\right)}\right).$$

The final anomaly score is defined as

(12) $$A^{\left(k\right)}\left(x\right)=\alpha_{\inf}\left(1-S_{s}\left(x\right)\right)+\left(1-\alpha_{\inf}\right)\left(1-S_{p}^{\left(k\right)}\left(x\right)\right),$$

which combines the global and client-specific similarity measures through a convex combination. The score is thresholded using a client-specific value $\rho^{\left(k\right)}$ selected from the calibration set to enforce the desired false-positive rate.

### 4.8. Optimization and Inference Procedures

#### 4.8.1. Federated Training Procedure

Algorithm 1 summarizes the federated optimization workflow of Fed-DTCN. The server initializes the global shared encoder parameters $\Theta_{s}^{0}$ and coordinates training over *R* communication rounds. At each round *r*, a subset of clients $S_{r}$ is selected, and the current shared parameters $\Theta_{s}^{r}$ are broadcast.
**Algorithm 1** Fed-DTCN Federated Training Procedure**Require:** Clients C; global rounds *R*; local epochs *E*; batch size *B*; augmentation family T; frozen auxiliary projector $\phi_{\mathrm{aux}}$; learning rate η.**Ensure:** Global shared encoder $\Theta_{s}$; local private encoders ${\left\{\theta_{p}^{\left(k\right)}\right\}}_{k=1}^{K}$.*       // Server-side initialization*     1:Initialize global shared parameters $\Theta_{s}^{0}$     2:**for** round r=0,…,R−1 **do**     3:     Select participating clients $S_{r}\subseteq C$     4:     Broadcast $\Theta_{s}^{r}$ to all $k\in S_{r}$*       // Parallel client updates*     5:      **for** each client $k\in S_{r}$ **do**     6:           $\Delta \theta_{s}^{\left(k,r\right)}\leftarrow$ ClientUpdate$\left(k,\Theta_{s}^{r}\right)$     7:      **end for***       // Weighted aggregation*     8:      $N_{\mathrm{round}}\leftarrow \sum_{j\in S_{r}}n_{j}$     9:      $\Theta_{s}^{r+1}\leftarrow \Theta_{s}^{r}+\sum_{k\in S_{r}}\frac{n_{k}}{N_{\mathrm{round}}}\Delta \theta_{s}^{\left(k,r\right)}$   10:**end for**   11:**return**$\Theta_{s}^{R}$   12:**function** ClientUpdate($k,\Theta_{s}^{\mathrm{global}}$)   13:      Load shared encoder $\theta_{s}^{\left(k\right)}\leftarrow \Theta_{s}^{\mathrm{global}}$   14:      Load private encoder $\theta_{p}^{\left(k\right)}$   15:      **for** epoch e=1,…,E **do**   16:            **for** mini-batch $B\subset D_{k}$ **do***       // Step 1: Semantic-aware view generation*   17:                 $\left(x_{1}^{\prime },x_{2}^{\prime }\right)∼T\left(B\right)$   18:                 Flatten views into V={1,…,2B}*       // Step 2: Auxiliary affinity estimation*   19:                  $e_{t}\leftarrow \phi_{\mathrm{aux}}\left(x_{t}^{\prime }\right),\forall t\in V$   20:                  Compute soft weights $w_{i\ell }$ using Equation (5)*       // Step 3: Forward pass*   21:                   $h_{s}\leftarrow E_{s}\left(V\right),\;h_{p}\leftarrow E_{p}\left(V\right)$   22:                   $z_{s}\leftarrow g_{s}\left(h_{s}\right),\;z_{p}\leftarrow g_{p}\left(h_{p}\right)$   23:                   ${\tilde{z}}_{s,t}\leftarrow z_{s,t}/{∥z_{s,t}∥}_{2},\;\forall t\in V$*       // Step 4: Optimization*   24:                 $L_{\mathrm{contrast}}\leftarrow L_{\mathrm{shared}}+L_{\mathrm{private}}$   25:                 Construct centered matrices ${\bar{H}}_{s},{\bar{H}}_{p}$   26:                 $L_{\mathrm{orth}}\leftarrow \frac{1}{d_{s}d_{p}}{∥{\bar{H}}_{s}^{⊤}{\bar{H}}_{p}∥}_{F}^{2}$   27:                 $L_{\mathrm{total}}\leftarrow L_{\mathrm{contrast}}+\lambda_{\mathrm{orth}}L_{\mathrm{orth}}$   28:                 Update $\theta_{s}^{\left(k\right)},\theta_{p}^{\left(k\right)}$ using Adam   29:           **end for**   30:      **end for**   31:      **return** $\theta_{s}^{\left(k\right)}-\Theta_{s}^{\mathrm{global}}$   32:**end function**

Each participating client executes a local ClientUpdate routine and returns only the shared-parameter update $\Delta \theta_{s}^{\left(k,r\right)}=\theta_{s}^{\left(k\right)}-\Theta_{s}^{r}$. Client-specific private parameters are never transmitted, ensuring that local variations remain isolated. The server aggregates updates using a sample-size-weighted average to obtain the next global model $\Theta_{s}^{r+1}$.

Within ClientUpdate, training proceeds using fully vectorized mini-batch operations. For each batch $B\subset D_{k}$, two semantic-preserving augmented views are generated per sample using the transformation family T. A frozen auxiliary projector $\phi_{\mathrm{aux}}$ maps the resulting views to an affinity space, from which pairwise distances and soft negative weights are computed. Shared and private encoders jointly process all views, and normalized projections are used to compute the corresponding contrastive objectives. An orthogonality regularizer is applied to explicitly disentangle global and client-specific representations. After *E* local epochs, only the shared-parameter update is returned to the server.

#### 4.8.2. Local Inference and Calibration

Algorithm 2 describes the client-side inference and decision-making procedure. During an offline calibration phase, each client computes branch-specific centroids using a held-out benign calibration set. These centroids characterize the expected representation of normal behavior in both the global and private embedding spaces.

At test time, each incoming window is encoded by the shared and private encoders, normalized, and compared to the corresponding centroids using cosine similarity. Global and local anomaly scores are computed independently and fused using a fixed mixing coefficient $\alpha_{\inf}$. The resulting anomaly score is thresholded using a client-specific decision threshold $\rho^{\left(k\right)}$. All calibration statistics remain local and may be refreshed periodically to accommodate slow changes in benign traffic patterns.
**Algorithm 2** Fed-DTCN Inference and Anomaly Detection**Require:** Test window $x_{\mathrm{test}}$; Trained encoders $E_{s},E_{p}$; Centroids $c_{s},c_{p}^{\left(k\right)}$; Mixing weight $\alpha_{\inf}$; Threshold $\rho^{\left(k\right)}$.**Ensure:** Anomaly Label y∈{0,1}.*       // Step 1: Feature Extraction*     1:$h_{s}\leftarrow E_{s}\left(x_{test};\theta_{s}^{\left(k\right)}\right)$     2:$h_{p}\leftarrow E_{p}\left(x_{test};\theta_{p}^{\left(k\right)}\right)$*       // Step 2: Normalization & Similarity*     3:${\hat{h}}_{s}\leftarrow h_{s}/∥h_{s}{∥}_{2},\;{\hat{h}}_{p}\leftarrow h_{p}/{∥h_{p}∥}_{2}$     4:$S_{s}\leftarrow {\hat{h}}_{s}^{⊤}c_{s}$                                                                                                *// Cosine similarity to global centroid*     5:$S_{p}\leftarrow {\hat{h}}_{p}^{⊤}c_{p}^{\left(k\right)}$ *// Cosine similarity to local centroid**       // Step 3: Score Fusion*     6:$Score_{global}\leftarrow 1-S_{s}$     7:$Score_{local}\leftarrow 1-S_{p}$     8:$A^{\left(k\right)}\leftarrow \alpha_{\inf}\cdot Score_{global}+\left(1-\alpha_{\inf}\right)\cdot Score_{local}$*       // Step 4: Decision*     9:**if** $A^{\left(k\right)}>\rho^{\left(k\right)}$ **then**   10:      **return** 1 *                                                                                                                     // Anomaly Detected*   11:**else**   12:      **return** 0 *                                                                                                                           // Benign Traffic*   13:**end if**

## 5. Performance Evaluation

### 5.1. Evaluation Datasets

Fed-DTCN is evaluated on two widely used intrusion detection benchmarks that represent complementary network environments: CSE–CIC–IDS2018 and TON_IoT. Table 3 reports the exact class labels and flow counts used consistently across all experiments to ensure reproducibility.

CSE–CIC–IDS2018 [32] is an enterprise-scale intrusion detection dataset released by the Communications Security Establishment and the Canadian Institute for Cybersecurity. It contains benign traffic alongside multiple attack categories, including brute-force and denial-of-service variants. For zero-day evaluation, the Bot attack class is entirely withheld from training and used exclusively during testing.

TON_IoT [33] is an industrial IoT dataset collected from a realistic IIoT testbed by the Australian Centre for Cyber Security. The network traffic portion includes normal activity and structured attack categories such as DoS, scanning, and injection. In this dataset, all attack classes are included during training, and no category is withheld.

While Table 3 details the global class distributions, the training regimes for the evaluated methods differ fundamentally. The supervised baseline (FeCo [6]) utilizes the full labeled training partition (benign and attack flows). In contrast, Fed-DTCN operates in a strictly unsupervised manner, accessing only the Normal/Benign samples during training to learn a nominal representation. For CSE–CIC–IDS2018, this creates an Open-Set challenge where the Bot class is entirely withheld from the training pool. For ToN_IoT, although attack classes are available in the training partition for supervised methods, Fed-DTCN treats them as out-of-distribution entities and trains solely on benign telemetry. This setup ensures that Fed-DTCN is evaluated under the most stringent conditions relative to supervised alternatives.

### 5.2. Global Preprocessing Pipeline

The preprocessing pipeline transforms heterogeneous raw network traffic into a standardized feature representation suitable for federated contrastive learning. Categorical features are partitioned based on cardinality using a threshold of 50 unique values. Low-cardinality attributes undergo One-Hot Encoding, while high-cardinality features (e.g., URI strings and DNS queries) are processed using a Robust Feature Hasher with 64 output dimensions to prevent dimensionality explosion. Missing values are handled via median imputation for numeric features and a constant MISSING token for categorical entries.

To ensure that the model learns an uncontaminated baseline of normal behavior, numeric features are standardized using statistics computed exclusively from benign training samples:

(13) $$x_{\mathrm{std}}=\frac{x-\mu_{\mathrm{benign}}}{\sigma_{\mathrm{benign}}}$$

This benign-only normalization strategy prevents anomaly-induced distribution shifts from influencing the normalization parameters, ensuring that anomalous samples remain detectable as statistical deviations. The resulting feature vectors are subsequently passed to the semantic-aware augmentation module and dual-encoder representation learning framework.

### 5.3. Federated Data Partitioning and Heterogeneity

**Figure 4:**
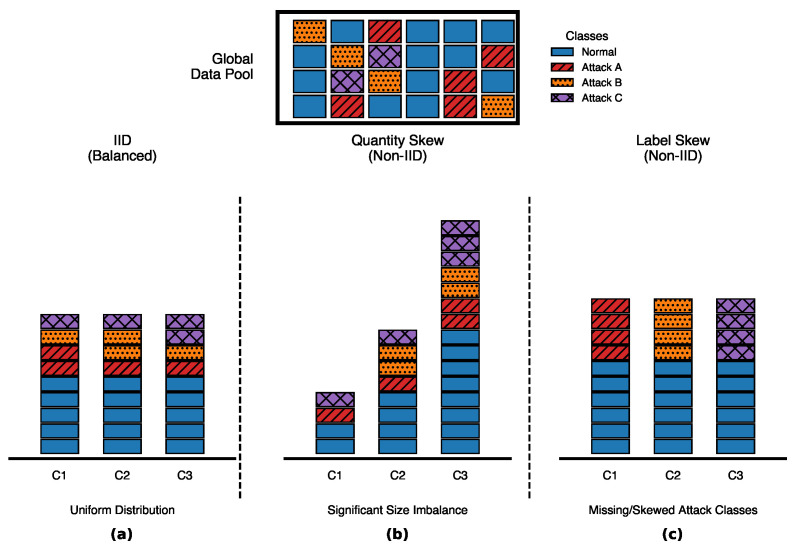
Federated data partitioning scenarios representing diverse IoT network conditions. (**a**) Balanced: Uniform distribution where local class proportions mirror the global data pool. (**b**) Quantity Skew (Non-IID): Significant size imbalance among clients reflecting heterogeneous device sampling rates. (**c**) Label Skew (Non-IID): Semantic heterogeneity characterized by missing or skewed attack classes across local partitions.

To simulate heterogeneous federated deployments, the global dataset is partitioned across K=10 clients under three distribution regimes: (i) an IID balanced split obtained by uniformly shuffling and evenly distributing the samples across clients, (ii) a non-IID quantity-skew split generated using a Dirichlet allocation over client sample counts, and (iii) a non-IID label distribution skew generated via class-wise Dirichlet sampling. These scenarios emulate federated environments where clients may exhibit different traffic volumes and observe different subsets of traffic classes (Figure 4).

#### 5.3.1. Scenario I: Statistical Homogeneity (IID)

An IID partition is constructed by uniformly shuffling D and allocating N/K samples to each client. This ensures that each client’s empirical class distribution $P_{k}\left(y\right)$ closely approximates the global prior $P_{\mathrm{global}}\left(y\right)$ and serves as an upper-bound reference for convergence and detection performance.

#### 5.3.2. Scenario II: Quantity Skew (Resource Imbalance)

To model heterogeneous device sampling rates and storage capacities, client sample counts are generated using a Dirichlet proportion vector $q∼\mathrm{Dir}(\beta 1_{K})$, where β>0 controls concentration. Each sample is assigned to client *k* with probability $q_{k}$. This approach preserves global class proportions within each client while inducing severe volume imbalance. We evaluate β∈{0.1,0.5,1.0}.

#### 5.3.3. Scenario III: Label Distribution Skew

To simulate semantic heterogeneity, where devices observe disjoint subsets of the attack manifold, we employ class-wise Dirichlet allocation [34]. For each class c∈C, a distribution $p_{c}∼\mathrm{Dir}\left(\alpha 1_{K}\right)$ is sampled, and each sample of class *c* is assigned to client *k* with probability $p_{c,k}$. The concentration parameter α controls the severity of label skew, with α=0.1 inducing extreme fragmentation and α≥1.0 approaching balanced allocation.

### 5.4. Implementation and Hyperparameter Settings

The Fed-DTCN framework is implemented in PyTorch 2.6 and evaluated in a GPU-accelerated federated simulation environment. To ensure fair and reproducible comparison, all baselines and proposed variants share the same backbone architecture, optimization schedule, and random seed initialization.

For feature extraction, we employ a Temporal Convolutional Network (TCN) with causal dilated convolutions. The architecture uses dilation factors d∈{1,2,4,8} to capture multi-scale temporal dependencies while strictly preventing future information leakage. The contrastive learning module consists of a two-layer MLP that projects backbone embeddings into a latent projection space. Shared and private projection dimensions are set to $m_{s}=256$ and $m_{p}=128$, corresponding to backbone latent sizes of $d_{s}=128$ and $d_{p}=64$, respectively.

Optimization is performed using the Adam optimizer with a local learning rate of η=0.0003 and a batch size of B=256. All semantic augmentation parameters, including time-warp amplitudes, masking probabilities, and volumetric scaling ranges, follow the final configurations listed in Table 4. In the experiments, the federated system consists of K=10 simulated clients. All clients participate in each communication round ($S_{r}=C$), corresponding to a full-participation setting. Each client performs E=2 local training epochs per round using mini-batches of size B=256. Aggregation of the shared encoder parameters is performed once per communication round using the sample-size-weighted FedAvg scheme defined in Equation (10). Preliminary experiments indicated that model convergence stabilizes within approximately 12–15 communication rounds; therefore, we set R=15 as the termination criterion.

To ensure a fair and transparent comparison, the personalized supervised baseline FeCo [6] is evaluated under the same preprocessing and experimental protocol as Fed-DTCN. In particular, it uses the identical feature sets (77 features for CSE–CIC–IDS2018 and 43 features for TON_IoT) and the normalization procedures described in Section 5.2.

Hyperparameters for the personalized supervised baseline are initialized according to the configurations reported in the original FeCo study [6] and further refined using a held-out validation split from the training data. To preserve realistic data characteristics, we retain the original dataset distributions and do not introduce additional resampling or synthetic balancing techniques. This unified experimental setup ensures that all methods are evaluated under the same preprocessing and data conditions.

### 5.5. Evaluation Metrics

All evaluation metrics are computed at the sliding-window level. Each input window x is treated as an independent decision instance and assigned a binary label, where the positive class corresponds to malicious activity and the negative class corresponds to benign traffic.

Let TP denote the number of attack windows correctly identified as malicious, TN the number of benign windows correctly classified as normal, FP the number of benign windows incorrectly flagged as attacks, and FN the number of attack windows that are missed by the detector. These quantities form the basis of all reported metrics.

- Accuracy: Measures the proportion of correctly classified windows over the total number of windows:
  (14) $$\mathrm{Accuracy}=\frac{TP+TN}{TP+TN+FP+FN}.$$
- Precision: Also known as positive predictive value, it quantifies the reliability of attack alarms:
  (15) $$\mathrm{Precision}=\frac{TP}{TP+FP}.$$
- Recall: Also referred to as sensitivity, it measures the fraction of actual attacks successfully detected:
  (16) $$\mathrm{Recall}=\frac{TP}{TP+FN}.$$
- F1-Score: The harmonic mean of precision and recall, providing a balanced view when classes are uneven:
  (17) $$F1=2\cdot \frac{\mathrm{Precision}\cdot \mathrm{Recall}}{\mathrm{Precision}+\mathrm{Recall}}.$$
- Area under the Precision–Recall Curve (PR-AUC): Measures the area under the precision–recall curve across all possible decision thresholds. PR-AUC is particularly informative for intrusion detection tasks with highly imbalanced data, as it focuses on the detector’s ability to correctly identify the positive (attack) class without being dominated by the large number of benign samples.
- Receiver Operating Characteristic (ROC-AUC): Represents the area under the ROC curve, which characterizes the trade-off between the true positive rate and false positive rate across decision thresholds. Although widely used, ROC-AUC may appear overly optimistic in severely imbalanced intrusion detection settings; therefore, it is interpreted together with PR-AUC and F1-score.

All metrics reported in Section 5.6 are computed on the held-out test data using the above definitions. In addition to threshold-dependent metrics, PR-AUC and ROC-AUC provide threshold-independent evaluation of the anomaly scores produced by the model. These metrics assess the ranking quality of anomaly scores across all possible decision thresholds, which is particularly important for intrusion detection scenarios characterized by highly imbalanced traffic distributions.

### 5.6. Comparative Analysis of Anomaly Detection Performance

We evaluate the anomaly detection performance of the proposed unsupervised Fed-DTCN framework against FeCo [6], a supervised federated contrastive learning baseline that relies on explicit attack labels during training. This comparison is intentionally stringent: FeCo [6] benefits from label supervision that is fundamentally unavailable to Fed-DTCN, thereby providing a strong reference point for detection performance on known attack categories. Although FeCo [6] is also designed as a personalized federated learning framework, the personalization mechanisms differ in their granularity. FeCo [6] introduces personalization through device-type grouping, where IoT devices are first categorized by type and separate federated models are trained for each device category. This strategy enables device-type-specific intrusion detection models but implicitly assumes that devices within the same category exhibit similar traffic distributions. In contrast, Fed-DTCN performs personalization at the individual client level through selective parameter aggregation. The proposed dual-encoder architecture disentangles shared and client-specific representations, where the shared encoder captures global traffic patterns and is aggregated across clients, while the private encoder remains local to each client. This design allows Fed-DTCN to preserve client-specific behavioral characteristics while still benefiting from collaborative knowledge sharing across the federation. Moreover, this client-level personalization allows Fed-DTCN to adapt to heterogeneous client traffic distributions without requiring predefined device-type groupings.

**Figure 5:**
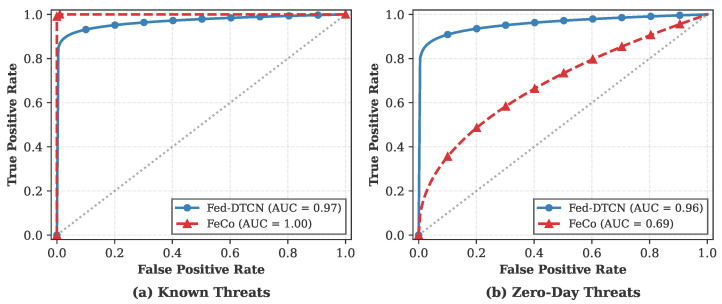
ROC curves comparing Fed-DTCN and FeCo [6] under (**a**) standard attack conditions and (**b**) zero-day attack conditions. While both methods perform comparably on known attacks, only Fed-DTCN maintains strong discriminative capability under open-set evaluation.

To ensure the statistical reliability of the reported results, we conducted multiple experimental runs using three different random seeds (42, 123, and 1046). Table 5 summarizes the detection performance on the TON_IoT dataset, reporting the mean and standard deviation for each metric. The results indicate that both Fed-DTCN and the supervised baseline exhibit high stability, with Fed-DTCN consistently achieving near-saturated performance across all independent trials. We analyze performance under two complementary regimes: detection of attacks observed during training and generalization to previously unseen (zero-day) threats. Threshold-dependent metrics are reported in Table 6, while threshold-independent behavior is examined using ROC curves in Figure 5.

#### 5.6.1. Performance on Standard IoT Traffic (TON_IoT)

We first report results on the TON_IoT dataset, which represents a relatively stationary IoT environment characterized by structured and recurring attack patterns. This setting evaluates the ability of Fed-DTCN to learn a discriminative representation of benign traffic in the presence of predictable attack behavior. As shown in Table 6, both approaches achieve near-saturated detection performance. Fed-DTCN attains a marginally higher F1-Score (99.99% vs. 99.34%), indicating that an unsupervised contrastive model of benign traffic—constructed using semantic-aware augmentations and dual-encoder disentanglement—can match the discriminative capability of a supervised approach when test-time attacks closely resemble those observed during training.

The ROC curves in Figure 5a indicate that both models operate near the optimal region when evaluated on known attacks. This narrow gap suggests that learning a robust representation of benign traffic through semantic-aware contrastive objectives can provide detection capability comparable to supervised approaches when the test distribution aligns with training conditions.

#### 5.6.2. Robustness Under Open-Set Conditions (CSE-CIC-IDS2018)

To evaluate zero-day detection capability, we adopt an open-set protocol on the CSE–CIC–IDS2018 dataset. The entire Botnet attack category is excluded from the training data and reserved exclusively for testing, ensuring that the model does not observe this attack type during representation learning. Training is performed in a strictly unsupervised benign-only regime. After training, anomaly detection thresholds are calibrated using a held-out benign calibration subset that is disjoint from both the training and testing data. Each client computes anomaly-score statistics on this benign subset and selects a local threshold $\rho^{\left(k\right)}$ to satisfy a target false-positive rate on benign traffic. No attack samples or labels are used during threshold calibration; all attack classes, including the withheld Botnet category, are introduced only during testing.

Known (Volumetric) Attack Classes. For attack categories observed during training, both methods achieve high recall, indicating effective detection of high-intensity volumetric threats. Fed-DTCN attains higher precision (94.72% vs. 87.78%), yielding an improved F1-Score of 96.85%. FeCo [6] achieves a higher PR-AUC (99.99% vs. 93.33%), which is consistent with supervised optimization toward labeled attack classes. In contrast, Fed-DTCN assigns anomaly scores based on deviation from a learned benign manifold, which may be more conservative for attacks whose characteristics partially overlap with normal high-volume traffic.

Zero-Day (Stealthy) Attack Detection. Under the zero-day evaluation protocol, the supervised FeCo [6] model exhibits a pronounced performance collapse (Recall: 0.28%, F1-Score: 0.52%), indicating limited generalization to the held-out Botnet category. This outcome reflects decision boundaries that are closely coupled to observed attack semantics.

In contrast, Fed-DTCN preserves strong detection capability, achieving an F1-Score of 96.00% and a PR-AUC of 89.16%. The ROC curves in Figure 5b show that Fed-DTCN maintains a steep ascent, whereas FeCo [6] exhibits near-random discrimination. These results indicate that unsupervised modeling of benign traffic dynamics yields superior robustness to unseen attack families.

### 5.7. Robustness to Data Heterogeneity and Zero-Day Threats

In realistic federated IoT deployments, client data are rarely IID. To evaluate robustness under statistical heterogeneity, we benchmark Fed-DTCN under IID, Quantity Skew (β=0.5,0.1), and Label Skew (α=0.1) partitioning schemes.

#### 5.7.1. Impact of Non-IID Distributions on Global Detection

**Figure 6:**
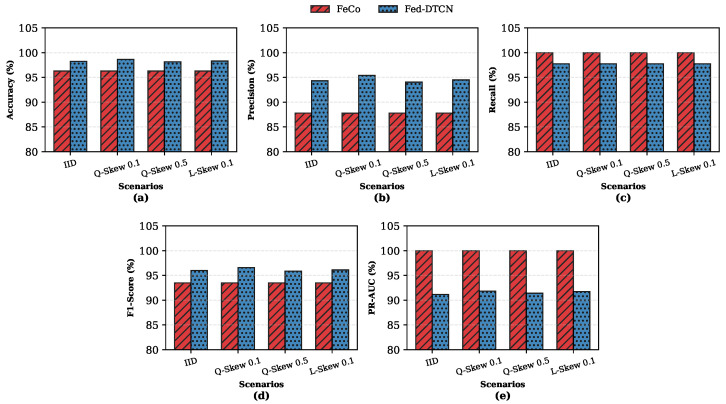
Global detection performance on known attacks (TON_IoT) under IID and non-IID data distributions: (**a**) Accuracy; (**b**) Precision; (**c**) Recall; (**d**) F1-Score; (**e**) PR-AUC. Fed-DTCN maintains stable F1-Scores across all skew scenarios, while the supervised baseline exhibits increased variability.

Figure 6 summarizes global detection performance on known volumetric attacks. Across all heterogeneity settings, Fed-DTCN demonstrates stable and consistently high performance. This robustness is attributable to the dual-encoder architecture, in which the shared encoder captures globally consistent benign patterns while private encoders absorb client-specific variability.

Under severe label skew (α=0.1), the supervised FeCo [6] baseline exhibits noticeable degradation, whereas Fed-DTCN maintains an F1-Score near 96%, indicating reduced sensitivity to class imbalance.

#### 5.7.2. Generalization to Zero-Day Threats Under Heterogeneity

**Figure 7:**
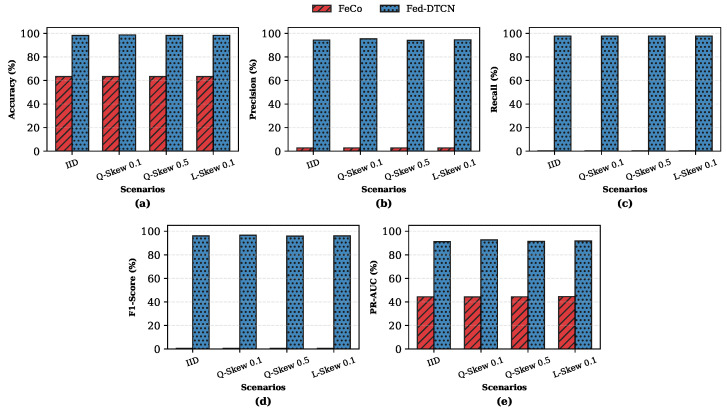
Zero-day detection performance (CSE-CIC-IDS2018 Botnet) under heterogeneous federated partitions: (**a**) Accuracy; (**b**) Precision; (**c**) Recall; (**d**) F1-Score; (**e**) PR-AUC. The supervised baseline fails to generalize, while Fed-DTCN preserves high detection accuracy across all skew levels.

Figure 7 shows that the supervised baseline exhibits severe generalization failure across non-IID settings. In contrast, Fed-DTCN maintains F1-Scores above 95% and recall exceeding 97% even under extreme quantity skew. Although PR-AUC values are moderately lower, such behavior is expected for stealthy attacks under class imbalance.

#### 5.7.3. Client-Level Stability and Personalization

**Figure 8:**
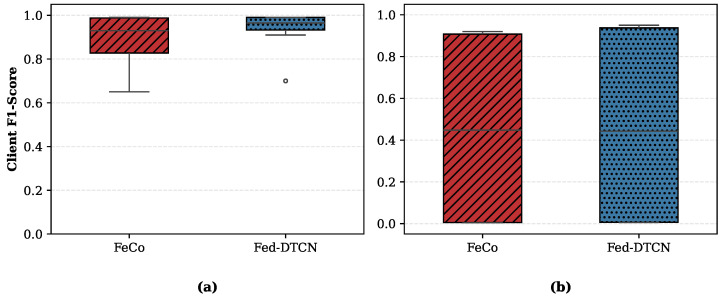
Client-level F1-Score distributions under different data regimes: (**a**) performance distribution under IID settings, where circles denote statistical outliers; (**b**) performance distribution under heterogeneous non-IID settings. Fed-DTCN exhibits significantly lower variance, indicating stable and uniform protection across heterogeneous clients.

Figure 8 illustrates client-level F1-Score distributions. Fed-DTCN produces tightly clustered scores with low variance, whereas the supervised baseline exhibits substantial inter-client variability. This stability is attributed to orthogonality regularization, which isolates client-specific variability while preserving globally shared benign structure.

**Figure 9:**
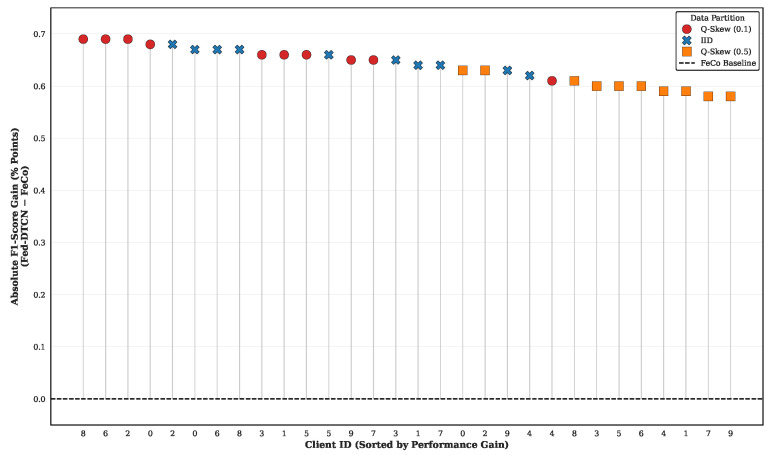
Client-level performance lift over FeCo [6]. All clients exhibit positive F1-Score gains, indicating the absence of negative transfer effects. The Y-axis represents the absolute gain (${F1}_{\mathrm{Fed}\text{-}\mathrm{DTCN}}-{F1}_{\mathrm{FeCo}}$).

Finally, to further characterize client-level personalization beyond variance-based analysis, we quantify the absolute performance lift achieved by the proposed unsupervised framework relative to a supervised federated baseline. Figure 9 reports the absolute F1-score gain, defined as ${F1}_{\mathrm{Fed}\text{-}\mathrm{DTCN}}-{F1}_{\mathrm{FeCo}}$, for each client, with clients sorted in descending order of improvement.

All participating clients exhibit strictly positive gains, with improvements ranging from approximately 0.58 to 0.70 absolute F1-score points. This consistent behavior holds across all data partitioning regimes, including IID and severe quantity-skewed settings, indicating that Fed-DTCN does not induce negative transfer for any individual client.

Taken together with the low inter-client variance observed earlier in this subsection, these results demonstrate that Fed-DTCN achieves personalization without sacrificing global consistency. The explicit disentanglement of shared and private representations enables local client characteristics to be absorbed by private encoders, while shared updates remain broadly beneficial across heterogeneous data distributions.

#### 5.7.4. Computational Complexity and Communication Overhead

To assess the practical feasibility of the proposed framework in federated IoT environments, we analyze both the model complexity and the communication overhead incurred during federated training. The computational complexity of the shared temporal convolutional encoder for an input sequence of length *T* can be approximated as

(18) $$O\;\left(\sum_{l=1}^{L}T\cdot k_{l}\cdot C_{l-1}\cdot C_{l}\right),$$

where *L* denotes the number of temporal convolution layers, $k_{l}$ represents the kernel size of layer *l*, and $C_{l}$ denotes the number of output channels. This formulation indicates that the computational cost of the encoder scales linearly with the sequence length, which is suitable for streaming IoT traffic analysis.

The complete Fed-DTCN architecture contains 6,277,536 parameters, corresponding to approximately 23.95 MB when stored using 32-bit floating-point representation. However, due to the selective aggregation mechanism described in Section 4.6, only the parameters of the shared encoder are transmitted between clients and the central server during each communication round. The shared encoder contains 4,023,808 parameters, representing 64.1% of the total model size.

Consequently, the communication payload per client per round is approximately 15.36 MB, while the remaining parameters belong to the client-specific private encoder and remain strictly local to each client. This design reduces the amount of data exchanged during federated training while allowing the model to maintain personalized representations for heterogeneous IoT environments.

Table 7 summarizes the model complexity and communication characteristics of Fed-DTCN and compares them with the supervised federated contrastive baseline FeCo. Although Fed-DTCN employs a larger backbone due to the temporal convolutional architecture and the dual-encoder design, selective aggregation ensures that only the globally shared parameters are exchanged during training. For a federation with *K* participating clients, the total uplink communication per round is approximately K×15.36 MB.

### 5.8. Ablation Study

To isolate and quantify the contribution of the proposed Semantic-Aware Causal Augmentation module, we conduct an ablation study under a challenging setting: zero-day detection on CSE-CIC-IDS2018 with severe data scarcity (Quantity Skew β=0.1). This configuration stresses both representation robustness and generalization under heterogeneous federated conditions.

We compare the full Fed-DTCN model against four degraded variants: (i) replacement of semantic augmentation with Gaussian noise, and (ii) systematic removal of individual semantic components—Time-Warping, Volumetric Scaling, and Protocol Masking.

#### 5.8.1. Effectiveness of Semantic-Aware Augmentation

Replacing semantic-aware augmentation with Gaussian noise produces a substantial degradation in detection performance, as detailed in Table 8. The F1-Score drops from 96.57% to 79.62%, accompanied by significant reductions in both Precision and Recall. These results indicate that unconstrained statistical perturbations disrupt the causal structure required to learn a coherent manifold of benign traffic behavior.

In addition to the augmentation analysis, we evaluate the contribution of the core architectural components of Fed-DTCN. As shown in Table 8, removing the private encoder (w/o Dual-Encoder) significantly reduces the F1-Score from 96.57% to 90.89%, indicating that separating shared and client-specific representations is critical for mitigating negative transfer across heterogeneous clients. Replacing the soft-weighted contrastive objective with a standard hard contrastive loss (w/o Soft-Weighting) further decreases the F1-Score to 89.29%, suggesting that adaptive weighting improves representation stability under non-IID conditions. Finally, replacing the TCN backbone with a standard CNN (w/o TCN) reduces performance to 90.29%, highlighting the importance of dilated temporal receptive fields for modeling the temporal dynamics of network traffic. These results confirm that each architectural component contributes independently to the overall effectiveness of Fed-DTCN.

**Figure 10:**
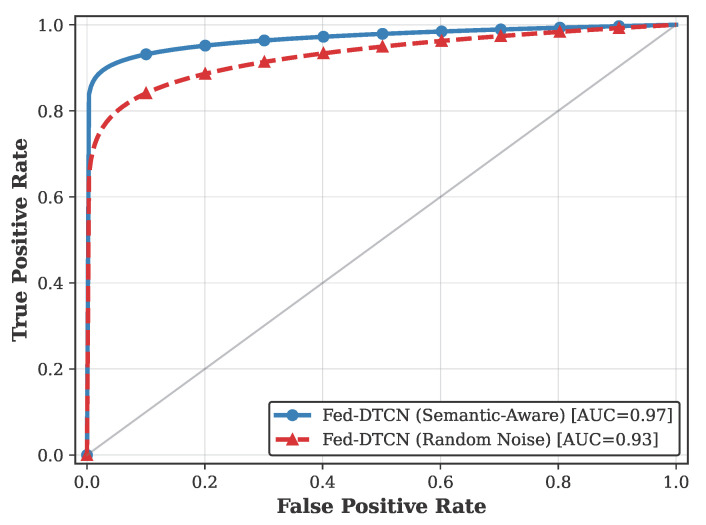
ROC curves for the ablation study under zero-day attack detection (CSE-CIC-IDS2018, Quantity Skew β=0.1). The full Semantic-Aware Fed-DTCN achieves the highest ROC-AUC.

The ROC curves in Figure 10 further confirm this observation. The full semantic-aware model achieves a ROC-AUC of 0.97, whereas the Random Noise variant exhibits a flatter trajectory with a ROC-AUC of 0.93, consistent with reduced discriminative power.

#### 5.8.2. Component-Level Analysis and Decision Boundary Behavior

Removing individual semantic components yields a consistent trade-off between sensitivity and precision. In all cases, Recall approaches saturation (>99.6%), while Precision decreases to approximately 81–82%, indicating a more permissive decision boundary. Each component enforces invariance along a specific dimension; its removal increases sensitivity to that dimension, causing benign fluctuations to be misclassified as anomalies.

The full Fed-DTCN model jointly enforces temporal, volumetric, and protocol-level invariances, achieving the best balance between sensitivity and false alarms, as reflected by the highest F1-Score, Accuracy, and PR-AUC.

### 5.9. Discussion and Analysis

The experimental results highlight a fundamental limitation of supervised federated intrusion detection approaches. While supervised baselines such as FeCo [6] perform well under stationary and closed-set conditions, their decision boundaries become brittle under distributional shift. When exposed to zero-day threats or heterogeneous client data distributions, performance degradation can be substantial.

#### 5.9.1. Decoupling Representation from Supervision

Fed-DTCN demonstrates that learning a representation of benign traffic dynamics provides improved robustness in open-world IoT settings. Benign traffic patterns tend to exhibit greater statistical consistency across clients than attack behaviors, enabling a federated model trained on normal activity to generalize more effectively than models optimized over skewed and incomplete attack labels.

#### 5.9.2. Structural Robustness to Heterogeneity

Client-level analysis further indicates that Fed-DTCN mitigates negative transfer effects commonly observed in supervised federated learning. Because local objectives are aligned around benign protocol dynamics rather than conflicting class labels, global aggregation remains stable even under severe non-IID conditions. This property is particularly important for large-scale IoT deployments, where client data distributions are inherently diverse and evolving.

#### 5.9.3. Semantic Constraints and Decision Boundary Formation

The ablation study clarifies the role of semantic constraints as more than a representational aid. Removing individual constraints—such as protocol-aware masking, temporal warping, or volumetric scaling—leads to near-saturated recall but substantially reduced precision, indicating an overly permissive notion of normality.

In contrast, the full Fed-DTCN configuration jointly enforces temporal, volumetric, and protocol-level invariances, yielding a more selective and operationally viable decision boundary. These constraints suppress benign variability while preserving sensitivity to stealthy deviations, highlighting their importance not only for contrastive learning but also for practical anomaly detection in heterogeneous IoT environments.

## 6. Conclusions and Future Work

This paper presented Fed-DTCN, a federated unsupervised framework for zero-day intrusion detection in IoT networks operating under statistical heterogeneity. By integrating a dual-encoder architecture with semantic-aware causal augmentations, the framework disentangles globally shared benign invariants from client-specific variations without reliance on labeled attack data. This design enables robust anomaly detection while preserving privacy and accommodating non-IID data distributions.

Extensive evaluation on standard and industrial IoT benchmarks demonstrates that Fed-DTCN matches supervised federated baselines on known attacks and substantially outperforms them under zero-day and heterogeneous conditions. The results indicate that modeling benign traffic dynamics provides a more stable and generalizable foundation for intrusion detection than supervised alignment to observed attack semantics.

Future work will explore several extensions of the proposed framework. First, we plan to investigate communication-efficient training strategies, including gradient compression and partial model aggregation, to further reduce bandwidth overhead in large-scale deployments. Second, we aim to extend Fed-DTCN to an online and continual learning setting to address gradual concept drift in benign traffic over long-term operation. Finally, incorporating lightweight adaptation mechanisms at the client level may further improve responsiveness to rapidly evolving local behaviors without compromising global model stability.

## Figures and Tables

**Table 1:** Comparison of representative intrusion detection and time-series anomaly detection approaches with respect to design dimensions related to the contributions of Fed-DTCN.

Method	Learning Paradigm	Architecture	Representation Learning	Representation Structure	Aggregation Strategy	Key Limitation for Federated Zero-Day IDS
FeCo [6]	Supervised contrastive learning	CNN encoder	Contrastive learning with labeled positives	Global shared representation	Global model aggregation in FL	Requires labeled attack samples, limiting applicability to benign-only zero-day detection scenarios
FADngs [8]	Self-supervised federated learning	Neural density estimator	Density-based representation learning with self-distillation	Global density representation	Exchange of density statistics and proxy distillation	Depends on representative proxy datasets and cross-client statistics exchange
FedCAD [9]	Federated anomaly detection	Graph embedding network	Graph-based embedding representations	Shared graph embeddings	Cross-client embedding exchange	Embedding sharing increases communication overhead and potential privacy exposure
FDFL [26]	Unsupervised anomaly detection	Autoencoder	Reconstruction-based representation learning	Shared and private feature decomposition	Aggregation of shared encoder	Reconstruction losses may incorporate anomalous patterns into the learned normal manifold
MGCLAD [30]	Contrastive anomaly detection	Graph neural network	Multi-view graph contrastive learning	Global graph representations	Centralized training	Requires explicit graph construction and centralized topology information
ContraMTD [22]	Graph contrastive learning	Graph encoder	Interaction-graph contrastive representations	Interaction graph embeddings	Centralized training	Depends on host interaction graphs unavailable in many practical IoT deployments
TS-TCC [20]	Self-supervised representation learning	Temporal CNN	Temporal prediction and permutation augmentations	Global temporal representation	Centralized training	Permutation operations disrupt packet ordering and protocol semantics in network traffic
TS2Vec [10]	Self-supervised representation learning	Dilated CNN	Hierarchical temporal contrastive learning	Hierarchical temporal representation	Centralized training	Generic augmentations may violate protocol semantics in network traffic
DCdetector [24]	Unsupervised anomaly detection	Transformer	Attention-based reconstruction learning	Global attention-based representation	Centralized training	Quadratic attention complexity limits deployment on resource-constrained edge gateways
MPFormer [23]	Unsupervised anomaly detection	Transformer	Patch-based temporal representations	Global patch-based representation	Centralized training	High computational and memory overhead for long time-series sequences
CARLA [12]	Contrastive anomaly detection	Deep neural encoder	Synthetic anomaly injection for contrastive learning	Global contrastive representation	Centralized training	Injected anomalies bias representations toward modeled attack patterns
Fed-DTCN (Ours)	Benign-only unsupervised learning	Temporal Convolutional Network (TCN)	Protocol-aware, causality-preserving augmentations	Explicit shared/private representation disentanglement	Selective aggregation of shared encoder parameters	Communication-efficient federated learning that preserves protocol semantics and supports heterogeneous IoT deployments

**Table 2:** Summary of Main Notations.

Category	Symbol	Description
Data	$D_{k},D$	Local and Global datasets
	$x,x^{\prime }$	Original traffic window and augmented view
	$n_{k},N_{\mathrm{tot}}$	Local and Global sample counts
	$x_{\mathrm{id}},x_{f}$	Identity/header features and behavioral flow metadata
Architecture	$\Theta_{s},\theta_{s}^{\left(k\right)}$	Global shared parameters and their local instances
	$\theta_{p}^{\left(k\right)}$	Local private parameters for client *k*
	$E_{s},E_{p}$	Shared and Private TCN encoders
	$d_{s},d_{p}$	Latent dimensions ($d_{s}=128$, $d_{p}=64$)
	$m_{s},m_{p}$	Projection dimensions ($m_{s}=256$, $m_{p}=128$)
	$\hat{h}$	$\ell_{2}$-normalized backbone embedding
Loss	$w_{i\ell },w_{\min}$	Soft negative weight and clamp value
	$\tau_{I},\gamma_{sw}$	Affinity distance decay and scaling factor
	$\phi_{\mathrm{aux}}$	Frozen auxiliary projector
	$L_{\mathrm{shared}},L_{\mathrm{orth}}$	Global contrastive and disentanglement objectives
Inference	$c_{s},c_{p}^{\left(k\right)}$	Global and Local normalized benign centroids
	$A^{\left(k\right)},\rho^{\left(k\right)}$	Final anomaly score and detection threshold

**Table 3:** Exact class distribution used in experiments.

Dataset	Class Label	Flows	Used in Training
CSE–CIC–IDS2018	Benign	2,872,859	✓
	DoS-Hulk	461,912	✓
	FTP-BruteForce	193,360	✓
	SSH-Bruteforce	187,589	✓
	DoS-SlowHTTPTest	139,890	✓
	DoS-GoldenEye	41,508	✓
	DoS-Slowloris	10,990	✓
	Bot	286,191	withheld
	Total	4,194,300	
TON_IoT	normal	482,528	✓
	dos	1,809,635	✓
	scanning	1,582,642	✓
	injection	125,195	✓
	Total	4,000,000	

**Table 4:** Simulation Parameters and Hyperparameter Settings.

Category	Parameter	Value
Federated Setting	Participating Clients (*K*)	10
	Global Comm. Rounds (*R*)	15
	Partitioning Scenarios	IID, Dirichlet (α,β)
	Heterogeneity Parameters	β∈{0.1,0.5} (Qty), α=0.1 (Label)
Local Training	Local Epochs (*E*)	2
	Batch Size (*B*)	256
	Optimizer	Adam
	Learning Rate (η)	0.0003
Dual-Encoder TCN	Input Window Size (*T*)	100
	Input Features (*F*)	77 (CSE-CIC-IDS2018)/43 (TON_IoT)
	Kernel Size (*k*)	7
	Dilations (*d*)	[1,2,4,8]
	Shared Latent/Proj Dims	$d_{s}=128,m_{s}=256$
	Private Latent/Proj Dims	$d_{p}=64,m_{p}=128$
	TCN Layers (Shared/Private)	4/4
	Auxiliary Dim ($d_{\mathrm{aux}}$)	32
Contrastive Objective	Temperature (τ)	0.5
	Orthogonality ($\lambda_{\mathrm{orth}}$)	0.001
	Soft-Weight Scaling ($\gamma_{sw}$)	0.5
	Distance Decay ($\tau_{I}$)	0.5
	Weight Clamp ($w_{\min}$)	0.001
	Projection Heads (MLP)	shared: 128→256→256, private: 64→128→128
Augmentation	Time-Warp Strength	0.10
	Feature Mask Prob	0.02
	Jitter/Scale	0.05/[0.9,1.1]

**Table 5:** Comparative Analysis of Detection Capability on ToN_IoT Dataset (Mean ± Std. Dev. over 3 random seeds).

Dataset	Test Scenario	Method	Accuracy	Precision	Recall	F1-Score	PR-AUC
ToN_IoT	Standard Attacks	FeCo [6]	98.94 ± 0.02	98.68 ± 0.01	100.00 ± 0.01	99.34 ± 0.04	100.00 ± 0.00
		Fed-DTCN	99.98 ± 0.07	99.99 ± 0.09	99.98 ± 0.01	99.99 ± 0.04	100.00 ± 0.00

**Table 6:** Comparative Analysis of Detection Capability on CSE-CIC-IDS 2018 Standard and Zero-Day Threats.

Dataset	Test Scenario	Method	Accuracy	Precision	Recall	F1-Score	PR-AUC
CSE-CIC-IDS2018	Known (Volumetric)	FeCo [6]	96.29	87.78	99.99	93.49	99.99
		Fed-DTCN	97.69	94.72	99.08	96.85	93.33
	Zero-Day (Stealthy)	FeCo [6]	63.36	2.75	0.28	0.52	44.29
		Fed-DTCN	98.24	94.32	97.74	96.00	89.16

**Table 7:** Model Complexity and Communication Overhead per Federated Round.

Method	Total Params	Model Size	Shared Params	Payload/Client	Bandwidth Reduction
FeCo [6]	93,952	0.36 MB	93,952 (100%)	0.36 MB	0%
Fed-DTCN	6,277,536	23.95 MB	4,023,808 (64.1%)	15.36 MB	35.9%

**Table 8:** Ablation Study of Semantic Augmentation and Architectural Components (Zero-Day Attack, Quantity Skew β=0.1).

Configuration	Strategy/Component	Accuracy	Precision	Recall	F1-Score	PR-AUC
Fed-DTCN (Full)	Proposed Framework	98.65	95.42	97.75	96.57	91.62
Augmentation Ablations						
w/o Random Noise	Gaussian Noise	89.00	78.49	80.79	79.62	85.78
w/o Time-Warping	Volumetric + Protocol	93.69	81.02	99.61	89.36	88.72
w/o Volumetric	Time + Protocol	93.82	81.32	99.65	89.56	89.56
w/o Protocol	Time + Volumetric	94.23	82.34	99.70	90.19	90.17
Architectural Ablations						
w/o Dual-Encoder	Shared Encoder Only	93.54	85.49	97.00	90.89	90.85
w/o Soft-Weighting	Hard Contrastive Loss	92.56	82.69	97.01	89.29	90.38
w/o TCN Backbone	Standard CNN	93.18	84.43	96.99	90.29	88.65

## Data Availability

Publicly available datasets were analyzed in this study. The details and citations for these datasets are provided in the article.
